# Rhein Protects Against Severe Acute Pancreatitis *In vitro* and *In vivo* by Regulating the JAK2/STAT3 Pathway

**DOI:** 10.3389/fphar.2022.778221

**Published:** 2022-03-17

**Authors:** Xiaofang Yang, Huan Geng, Lijiao You, Lin Yuan, Jialei Meng, Yuhui Ma, Xuelian Gu, Ming Lei

**Affiliations:** Department of Critical Care Medicine, Seventh People’s Hospital Affiliated to Shanghai University of Traditional Chinese Medicine, Shanghai, China

**Keywords:** rhein, severe acute pancreatitis, JAK2/STAT3, TNF-α, IL-6

## Abstract

Rhein is widely used in inflammation treatment in China, but its effects on severe acute pancreatitis (SAP) have not been studied closely. This study investigated rhein’s protective effects against SAP using *in vitro* and *in vivo* models to determine whether its protective mechanism regulated the Janus kinase two and signal transducer and activator of transcription 3 (JAK2/STAT3) signalling pathway. Thirty-six male Sprague–Dawley rats were randomised into sham operation, SAP and rhein groups. The SAP model was induced by retrograde pancreatic bile duct injection of sodium taurocholate. Serum TNF-α and interleukin (IL)-6 levels were determined by ELISA, whereas serum amylase and lipase concentrations were measured using test kits. Western blot and/or immunohistochemistry quantified JAK2 and STAT3 expression. Furthermore, histopathological pancreatic changes were detected by haematoxylin and eosin staining. AR42J cells were randomly divided into the control, cerulein and rhein groups. Amylase activity was assessed using an amylase test kit; the tumour necrosis factor-*α* (TNF-α) expression was determined by enzyme-linked immunosorbent assay (ELISA). JAK2 and STAT3 protein expression were evaluated by western blot. SAP was concomitant with increased JAK2 and STAT3 expressions *in vivo*. Pre-treatment with rhein attenuated serum TNF–α and IL-6 levels effectively, and notably reduced p-JAK2, p-STAT3, JAK2 and STAT3 protein expression. Rhein significantly alleviated pancreatic histopathology. Compared to untreated groups, rhein significantly reduced amylase activity in supernatants of AR42J cells induced by cerulein *in vitro*. Furthermore, rhein altered JAK2 and STAT3 protein levels in AR42J cells after cerulein induction. Overall, rhein exerted protective effect on SAP *in vitro* and *in vivo*, possibly through the JAK2/STAT3 signalling pathway.

## Introduction

Acute pancreatitis is an acute inflammatory disease that severely affects health and threatens life, and about 30% of patients with acute pancreatitis progress to severe acute pancreatitis (SAP) (Lankisch and Lerch, 2006; Morel et al., 2006). About 15%–20% of SAP cases often develop multiple systemic complications, such as the liver, intestine, kidneys and lungs complications, and the therapeutic effects of current treatments are unsatisfactory (Zerem, 2014).

Some studies propose that the effective inhibition of inflammation is key to preventing SAP progression (Gallmeier et al., 2005; Andersson et al., 2007; Ivanenkov et al., 2011). Therefore, cell signalling pathways related to inflammation in SAP have been the subject of increased research attention. The Janus kinase two and signal transducer and activator of transcription 3 (JAK2/STAT3) signalling pathway, one of the main cytokine signalling pathways, has become a hotspot of pancreatic research in recent years. The JAK2/STAT3 signalling pathway is widely involved in disease physiology and pathogenic development, including intracellular homeostasis, immune response, cell proliferation and apoptosis (O’shea, 2004). Tumour necrosis factor-*α* (TNF-α) and interleukin (IL)-6 are important transduction factors in the JAK2/STAT3 signalling pathway that can induce the inflammatory cascade, enhance the inflammatory effect and even lead to organ damage and multiple organ dysfunction syndrome in SAP (Zhu et al., 2017). Moreover, the JAK2/STAT3 signalling pathway amplifies the inflammatory response by initiating a series of inflammatory factor transmissions and related protein expression, thereby creating a waterfall effect. Several previous studies have shown that the JAK2/STAT3 pathway plays a key role in inflammatory diseases (Zhu et al., 2017; Xia et al., 2021). Thus, inhibiting this pathway may restrain the expansion of early cascading inflammatory response and prevent acute inflammatory injury to related tissues.

The current treatment protocol for SAP is a multi-targeted therapy based on the diagnostic and treatment guidelines for SAP, which focuses on pharmacological blockade of necrosis, inflammation and cellular damage in the pancreas. The multi-targeted action of natural drugs has attracted attention, compared to the single-targeted action of western drugs (Xue et al., 2006; Weng et al., 2012; Xia et al., 2012; Zhong, 2015). Rhein is also a natural molecule and is widely found in medicinal plants such as rhubarb, *Sennae folium*, *Semen cassiae*, and *Polygonum multiflorum*, and it is widely used in clinical practice (Xian et al., 2020). In China, approximately 10% (800) of more than 8,000 proprietary Chinese medicines contain rhubarb.

**FIGURE 1:**
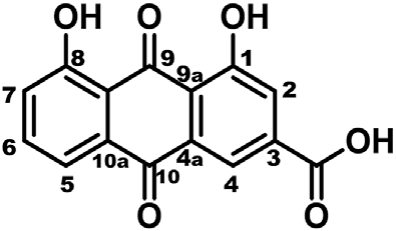
The chemical structure of rhein.

Rhein (the chemical structure is shown in Figure 1, the main rhubarb component, is reported to be widely used in inflammation treatment in China (Cheng et al., 2021). Rhein is also the major active component in Dachengqi Decoction with strong anti-inflammatory effects (Xu et al., 2008; Xu et al., 2010; Xu et al., 2010). Several studies have shown that Dachengqi Decoction can improve the inflammatory response in patients with SAP and reduce pathological pancreatic damage (Chen et al., 2010; Chen et al., 2015). In addition, the JAK2/STAT3 is predicted to be one of the targets of rhein according to network pharmacology. While rhein has been shown to play a prominent role as an inhibitor of the JAK2/STAT3 pathway in several experimental models and human diseases (Zhang et al., 2016; Yang et al., 2019). However, it is unclear whether rhein has a therapeutic effect on SAP by inhibiting JAK2/STAT3 pathway. Therefore, the present study was designed to investigate rhein’s protective effects on SAP using *in vitro* and *in vivo* models.

## Materials and Methods

### Drugs and Reagents

Purified rhein (Cat. No. 478-43-3) was purchased from the National Institute for Food and Drug Control (Beijing, China), the purity by authorization >98%. Sodium taurocholate (Cat. No. T4009), cerulein (Cat. No. C9026) and 5% carboxymethylcellulose sodium (CMCS) (CAS number:9004-32-4) were purchased from Sigma-Aldrich Merck KGaA (Darmstadt, Germany). Amylase kits (Cat. No. C016-1-1) were purchased from Nanjing Jiancheng Institute of Bioengineering (Jiangsu, China). Foetal bovine serum (Cat. No.10099-141), F12K medium (Cat. No. C11330500BT) and trypsin (Cat. No. 25200-056) were purchased from Gibco Ltd. (United States). PCR kits (Cat. No. RR820Q) were purchased from TaKaRa Corporation (Japan). GoScript Reverse Transcription Kits (Cat. No. A5001) were purchased from Promega (United States). Phosphatase inhibitors (Cat. No. P1081), SDS–PAGE gel preparation kits (Cat. No. P0012A) and ECL hypersensitive colour development kits (Cat. No. P0018AS) were purchased from Shanghai Biyuntian Biotechnology (Shanghai, China).

### Cellular Model

Rat pancreatic acinar AR42J cells were obtained from the American Type Culture Collection and were cultured in F12K medium (Cat. No. C11330500BT, Gibco) supplemented with 20% Australian foetal bovine serum (Cat. No. 10099141C, Gibco), 100 U/ml penicillin and 100 mg/l streptomycin (Cat. No. 15140-122, Gibco) at 37°C and 5% CO_2_. The AR42J cells were divided into three groups: control group, cerulein group (cerulein 10^–8^ mol/L) (Lee et al., 2003; Huang et al., 2012; Yu et al., 2003; Zhao et al., 2014; Chen et al., 2019) and rhein group (final concentration 0.25, 0.5 and 1 μg/ml). The AR42J cells density was 5×10^6^/ml. In the cerulein group, cells and culture supernatants were collected at 0, 0.5, 1, 2, 4, 6, 12 and 24 h after the addition of cerulein.

Animal Model Thirty-six adult male Sprague–Dawley rats (8–10 weeks old, weighing 200–250 g) were obtained from the Shanghai SLAC Laboratory Animal Co., Ltd. SD rats were randomly divided into three groups: sham operation group (n = 12), SAP model group (*n* = 12), rhein (dissolved in 0.5% CMCS) treatment group (*n* = 12). All rats were maintained under standardised conditions on a 12 h light/dark cycle and allowed free access to food and water. The rats fasted for 12 h before the operation, however, drinking water was available ad libitum. The SAP model was induced via a standardised pressure-controlled infusion of 3.5% sodium taurocholate (1 ml/kg, 0.1 ml/min) into the bile–pancreatic duct, and the common hepatic duct was closed with a small clamp for 5 min as previously described (Pérez et al., 2015). Subsequently, the clamp was removed, and the abdomen was closed. Rats in the sham control group underwent the same operative procedure with the injection of 0.9% saline in the pancreatic duct. In the rhein group, rats were given rhein by a gastric gavage (30 mg/kg, 0.5 ml/100 g) (Xian et al., 2020) once a day for 7 days prior to establishing the SAP model. Each group was orally administered with by gavage rhein (30 mg/kg) or saline for seven continuous days. All samples were collected 24 h after operation. All protocols and procedures were established in compliance with the US National Institute of Health Guidelines for the Care and Use of Laboratory Animals. They were approved by the Animal Care and Use Committee of Shanghai University of Traditional Chinese Medicine (Protocol number: PSHUTCM200103002).

Amylase, IL-6, TNF-α, lipase And myeloperoxidase Assays.

Amylase (Cat. No. C016-1-1), lipase (Cat. No. A054-2-1) and myeloperoxidase (Cat. No. A044-1-1) were purchased from Nanjing Jiancheng Institute of Bioengineering (Jiangsu, China). Serum amylase, lipase and myeloperoxidase concentrations were measured using test kits according to the manufacturer’s instructions. The amounts of TNF-α (Cat. No. E-EL-M0049c) and IL-6 (Cat. No. E-EL-R0015c) were determined using ELISA kits (eBioscience Ltd., United States) according to the manufacturer’s instructions.

### Western Blot

RIPA lysis buffer was added to pancreatic tissues and centrifuged at 13,700× *g* at 4°C for 15 min. Supernatants were harvested, and the BCA protein kit was used to determine each protein sample’s protein content. SDS–PAGE gels (10% separating gel, 3% concentrating gel) were prepared. After electrophoresis, the protein was transferred to a PVDF membrane, which was then sealed using 5% BSA for 2 h at room temperature. The following primary antibodies were added and incubated at 4°C overnight: JAK2 (Cat. No. 3230, 1:1,000, Cell Signaling Technology Inc.), STAT3 (Cat. No. 12640, 1:2000, Cell Signaling Technology Inc.), p-JAK2 (Cat. No. 3776, 1:1,000, Cell Signaling Technology Inc.), p-STAT3 (Cat. No. 9145, 1:1,000, Cell Signaling Technology Inc.) and GAPDH (Cat. No. 5174, 1:1,000, Cell Signaling Technology Inc). The membranes were then washed with Tris-Buffered Saline Tween buffer three times for 10 min and incubated with horseradish peroxidase (HRP)-labelled goat anti-rabbit IgG secondary antibody (Cat. No. 7074P2, 1:3,000, Cell Signaling Technology Inc.) for 2 h at room temperature. Afterwards, the membranes were washed with Tris-Buffered Saline Tween buffer three times for 10 min. Ultra-sensitive ECL luminescence solution was then added to detect the proteins, and a Fluorchem FC3 gel imager was used for detection. ImageJ 1.52a software was used to determine the gray value of the band. .

### Pathological Changes in the Pancreas

The rat pancreatic tissues were fixed in 4% paraformaldehyde and embedded in paraffin wax for routine sections. The sample was cut into 5 µm thick sections. After staining with haematoxylin and eosin, the sections were photographed under the microscope (Nikon FCI IPSE NI, Nikon Corporation) at ×200 magnification for image processing and analysis. Pancreatic histopathology was scored by the double-blind method (Schmidt et al., 1992). The mean total score of each histopathological variable was subsequently calculated.

The methods of histological score of pancreatic injury were as follows: Edema: 0, absent; 1, focal expansion of the interlobar septa; and 2, diffuse expansion of the interlobar septa. vascular: 0, absent; 1, congestion; 2, congestion plus local interlobar or intralobar hemorrhage; 3, multifocal diffuse hemorrhage; and 4, vascular fibrinoid necrosis or thrombosis. Fat necrosis: 0, absent; 1, focal dissolution of the interlobular or peripancreatic fat; and 2, diffuse dissolution of the interlobular or peripancreatic fat. Acinar necrosis: 0, absent; 1, <10% patchy necrosis of the edges of the lobules; 2, 10–30% patchy, peripheral necrosis of the lobules; 3, <30% confluent lobules necrosis; 4, <50% confluent lobular necrosis; and 5, formation of microabscesses. Calcification: 0, absent; 1, focal in fat or acinar necrosis; and 2, diffuse in fat or acinar necrosis.

### Immunohistochemistry

The rat pancreatic tissues slices were dewaxed with xylene, dehydrated with an ethanol gradient, and then placed in a sodium citrate buffer solution for 15 min. The slices were cooled for 30 min and then treated with 3% hydrogen peroxide for 10 min at room temperature. After three washing with PBS (phosphate buffer saline), shook off the PBS before adding antibody to each slice. The following antibodies were added and incubated at 4°C overnight: p-JAK2 (Cat. No. ab32101, 1:50, Abacam Company), p-STAT3 (Cat. No. ab267373, 1:250, Abacam Company), After three washings with PBS, the HRP-labelled goat anti-rabbit IgG secondary antibody was added and incubated at room temperature for 1 h. The tissue sections were then covered with DAB solution. After colour development, the sections were rinsed with water. Hematoxylin stained for 1 min 20 s, then soaked in tap water, alcohol fractionated in 1% hydrochloric acid for 1 s, soaked in tap water and rinsed under running water for 7–8 min, dried at 65° for 30 min, then taken out and cooled to room temperature and sealed in neutral gum. Image processing and photography, two different observers calculated the positive areas.

### Molecular Docking

Rhein was preprocessed by the Ligprep 3.6 program (Schrödinger, LLC, New York, NY, United States) applying OPLS_2005 force field before molecular docking, with Epik 3.4 (Schrödinger, LLC, New York, NY, United States) to generate the proper protonation states at pH 7.0 ± 2.0. A restrained minimization of the crystal structure was performed to reorient side-chain hydroxyl groups before further processing. The DQX pocket of 3KRR was selected to define and generate the receptor grid. In silico docking was performed by Glide 6.9 (Schrödinger, LLC, New York, NY, United States) in standard precision (SP) with default values for other parameters.

Statistical Analysis Statistical analyses of the obtained experimental data was carried out using SPSS version 21.0. ImageJ 1.52a software was used to calculate the greyscale value. Significance between groups was evaluated by one way analysis of variance (ANOVA) followed by a Tukey post hoc test. Data are expressed as mean ± SD, and *p* < 0.05 was considered statistically significant.

## Results

The network pharmacology results of rhein with JAK2 molecule.

**FIGURE 2:**
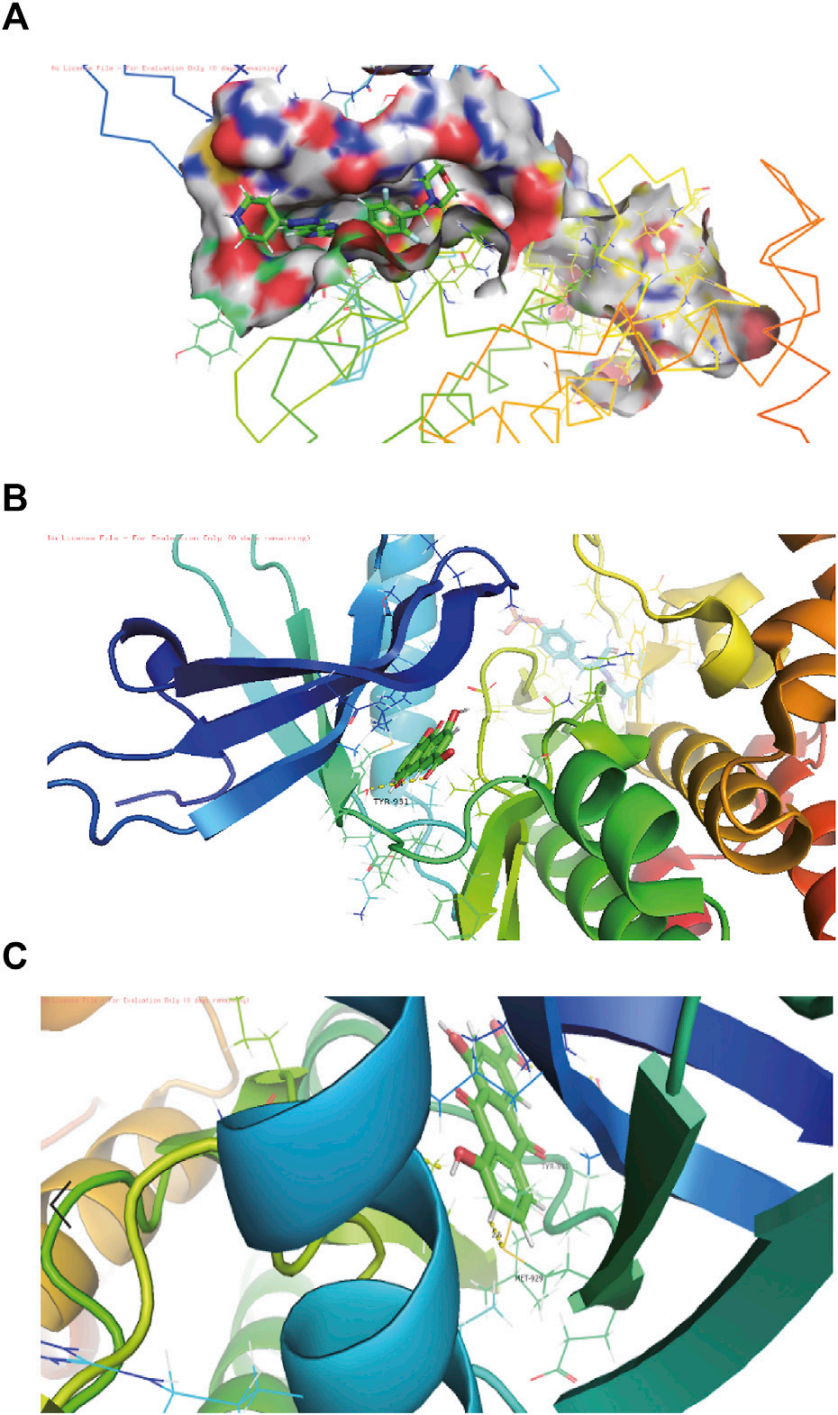
The structure-activity relationships of rhein with JAK2 molecule. **(A)** Schematic diagram of the connection between jAk2 and 3kRR in the software pymol view, 3kRR is a compound structure that can already bind to JAK2. **(B)** Schematic diagram of docking with the pocket after rhein is replaced by 3KRR through molecular docking. **(C)** The residues in the pocket TYR-931 and MET-929 are the residues in the pocket, which they are stable binding sites for rhein.

3KRR is a Crystal Structure of JAK2 complexed with a potent quinoxaline ATP site inhibitor (Figure 2A). The pocket factor of 3KRR was mostly buried in the hydrophobic pocket of DQX, and its main body forms abundant hydrophobic interactions with surrounding residues, whereas its head forms hydrogen bonds with residues nearby the entrance and middle position of the pocket. The in silico docking models showed that the binding sites of the rhein was located near the entrance and middle part of the 3KRR pocket, find rhein partially overlapping with that of the pocket factor of Tyrosine 931 (TYR-931) and MET-929 (Figure 2B, Figure 2C), they may play an important role in the interaction of compounds with pockets, by preempting residue positions and thus achieving the ability to inhibit other compounds from binding to them.

### The Effect of Rhein on Inflammatory Cytokines Levels in SAP Rats

**FIGURE 3:**
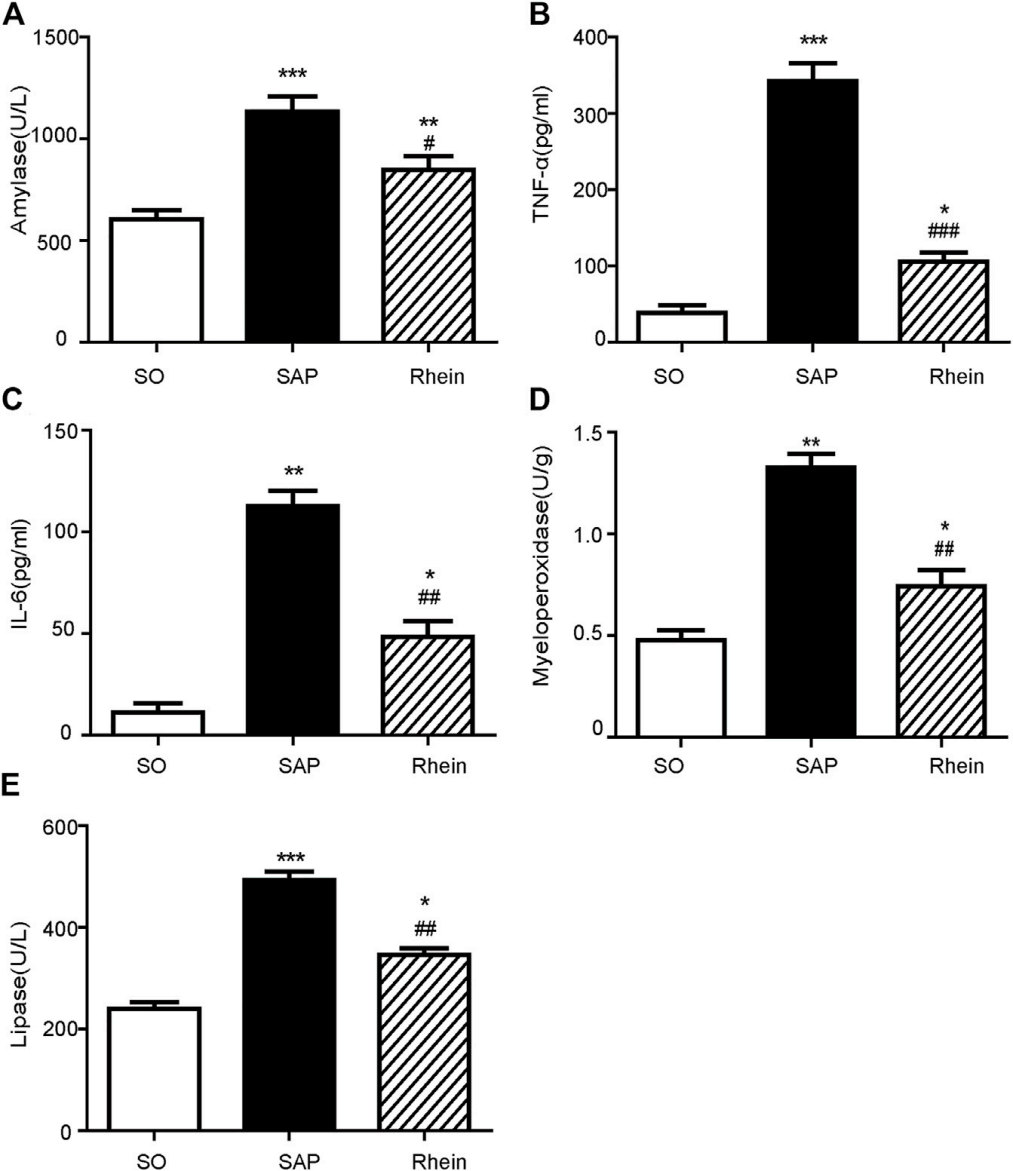
Effects of rhein on the inflammatory cytokine levels in pancreatic tissue of the SAP rat model. **(A)** Serum amylase levels in the SO, SAP and rhein groups. **(B)** Serum TNF-α levels in the SO, SAP and rhein groups. **(C)** Serum IL-6 levels in the SO, SAP and rhein groups. **(D)** Myeloperoxidase levels in the SO, SAP and rhein groups. **(E)** Serum lipase levels in the SO, SAP and rhein groups. Data are expressed as mean ± SD (*n* = 5 per group). Significance between groups was evaluated by one way analysis of variance (ANOVA) followed by a Tukey post hoc test. *p < 0.05, **p < 0.01 and ***p < 0.001 compared with the SO group; ^#^ *p* < 0.05, ^##^ *p* < 0.01 and ^###^ *p* < 0.001 compared with the SAP group. SO, sham operation; SAP, severe acute pancreatitis; TNF-α, tumour necrosis factor-*α*; IL-6, interleukin-6.

Figure 3 shows the effect of rhein on the level of inflammatory cytokines in pancreatic tissue of SAP rat model. Compared with the control group, the levels of serum amylase, serum TNF-α, serum IL-6, myeloperoxidase and serum lipase were significantly increased in the SAP group (*p* < 0.001). After rhein treatment, the levels of serum amylase, serum TNF-α, serum IL-6, myeloperoxidase and serum lipase were significantly decreased in the rhein group compared with the SAP group (*p* < 0.001).

### Effect of Rhein on Pathological Dmage in Pancreatic Tissue of SAP Rats

**FIGURE 4:**
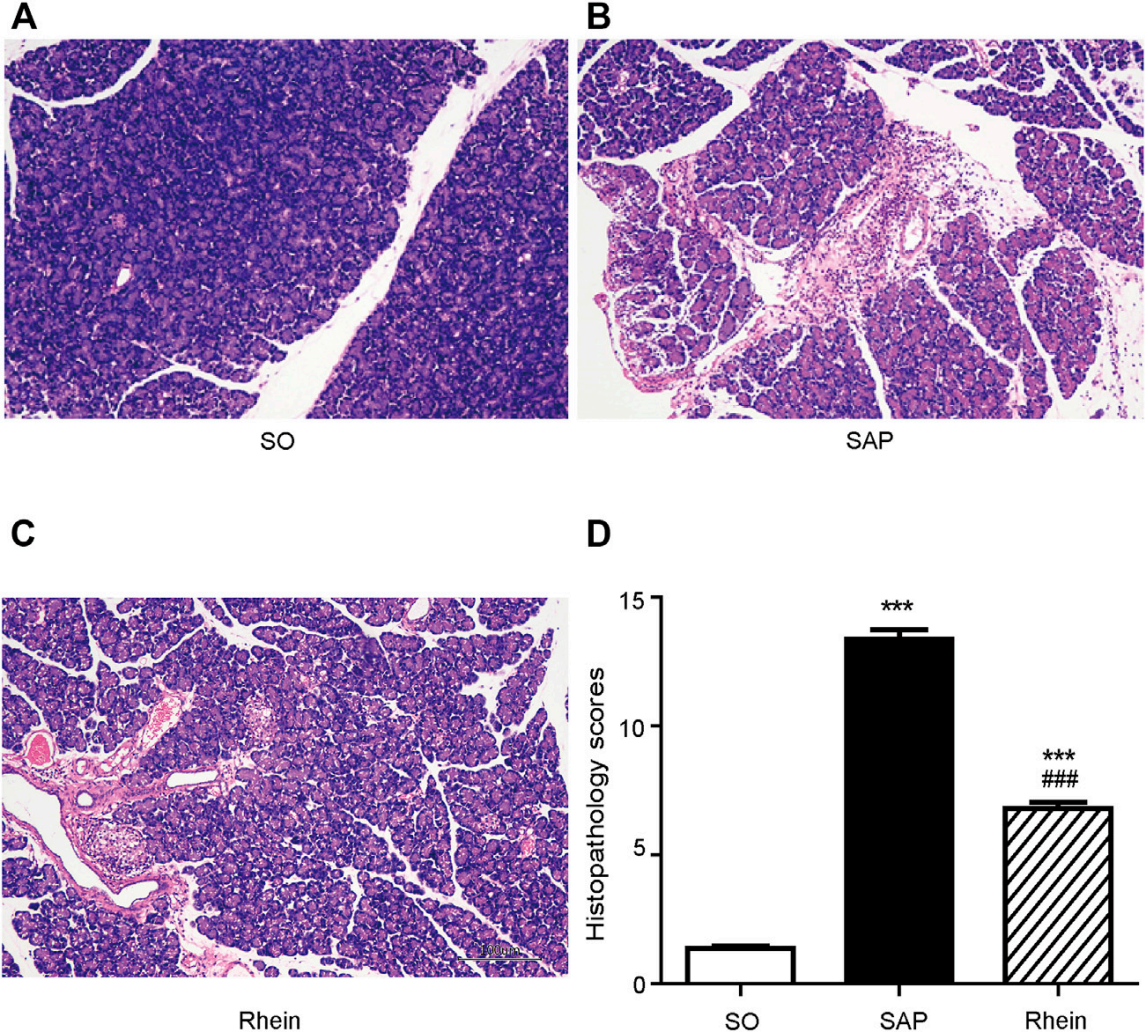
Histological analysis of SAP. (**A–C)** Morphological analysis of H&E-stained sections; Scale bar: 100 μm. **(D)** Pathological scores of pancreatic tissue sections. Data are expressed as mean ± SD (*n* = 3 per group). Significance between groups was evaluated by one way analysis of variance (ANOVA) followed by a Tukey post hoc test. *p < 0.05, **p < 0.01 and ***p < 0.001 compared with the SO group; ^#^ *p* < 0.05, ^##^ *p* < 0.01 and ^###^ *p* < 0.001 compared with the SAP group. SO, sham operation; SAP, severe acute pancreatitis; H&E, haematoxylin and eosin.

Figure 4 shows the protective effect of rhein on pancreatic injury. In the control group, typical normal acinar architecture were observed. Only minimal focal edema was detected, and no apparent inflammatory infiltration was found. Compared with the control group, edema, necrosis and inflammatory infiltration were observed in the acinar cells of pancreatic tissue in the SAP group. Compared with the SAP group, less infiltration of inflammatory cells was observed in the rhein group, Only fewer abnormal acinar cells were detected. Figure 4D reveals that the pathological scores were significantly increased in the SAP group compared with the control group (*p* < 0.01). Compared with the SAP group, the pathological scores were significantly decreased in the rhein group (*p* < 0.05).

### Effects of Rhein on p-JAK2, p-STAT3, JAK2 and STAT3 Expression in Pancreatic Tissue of SAP Rats

**FIGURE 5:**
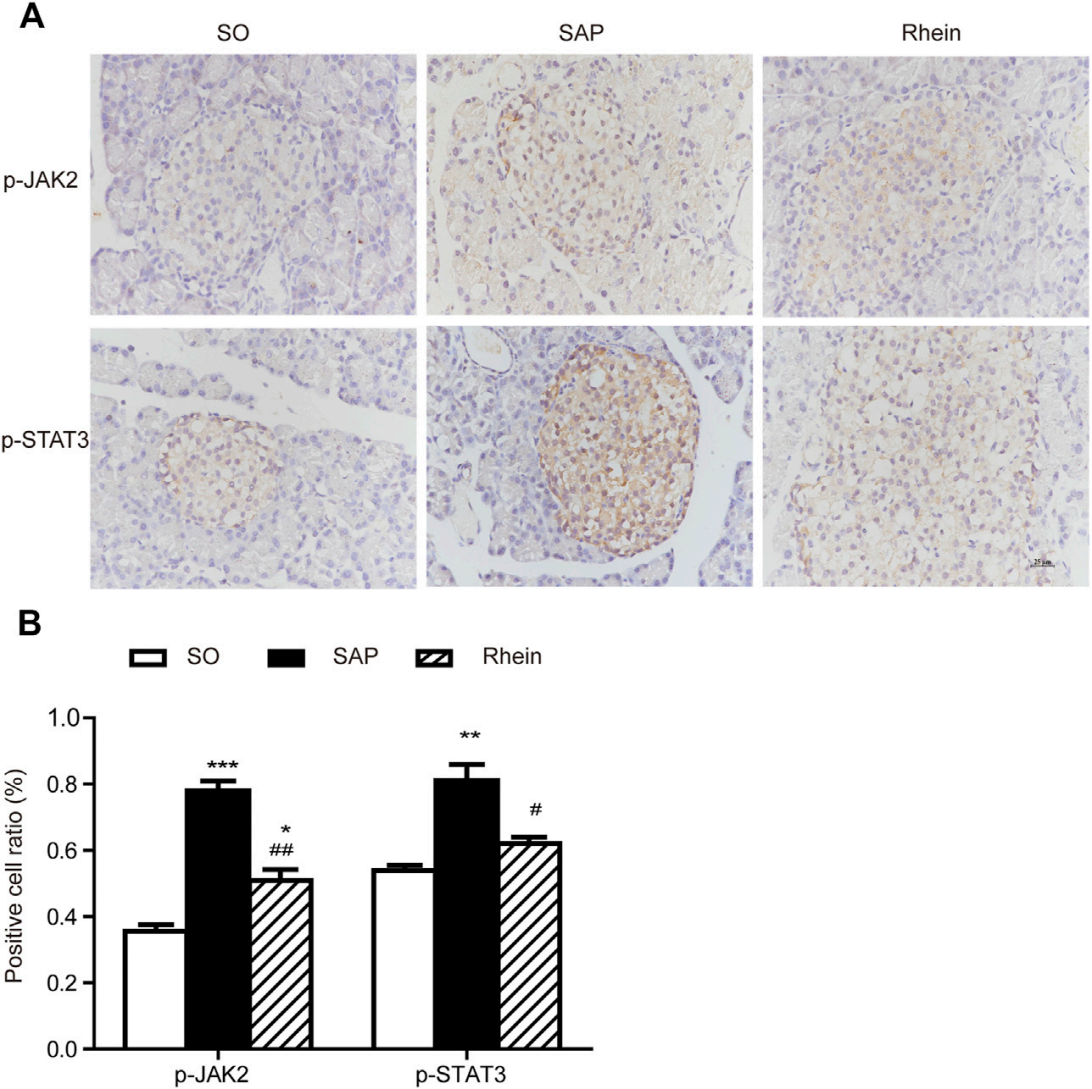
Immunohistochemical analysis of p-JAK2 and p-STAT3 in pancreatic tissues. **(A)** Representative immunohistochemistry images of p-JAK2 and p-STAT3 in pancreatic tissues 24 h after the induction of SAP; Scale bar: 25 μm. **(B)** Positive cell ratio of pancreatic tissue sections. Data are expressed as mean ± SD (*n* = 3 per group). Significance between groups was evaluated by one way analysis of variance (ANOVA) followed by a Tukey post hoc test. *p < 0.05, **p < 0.01 and ***p < 0.001 compared with the SO group; ^#^ *p* < 0.05, ^##^ *p* < 0.01 and ^###^ *p* < 0.001 compared with the SAP group. JAK2, janus kinase two; STAT3, signal transducer and activator of transcription three; SO, sham operation; SAP, severe acute pancreatitis.

**FIGURE 6:**
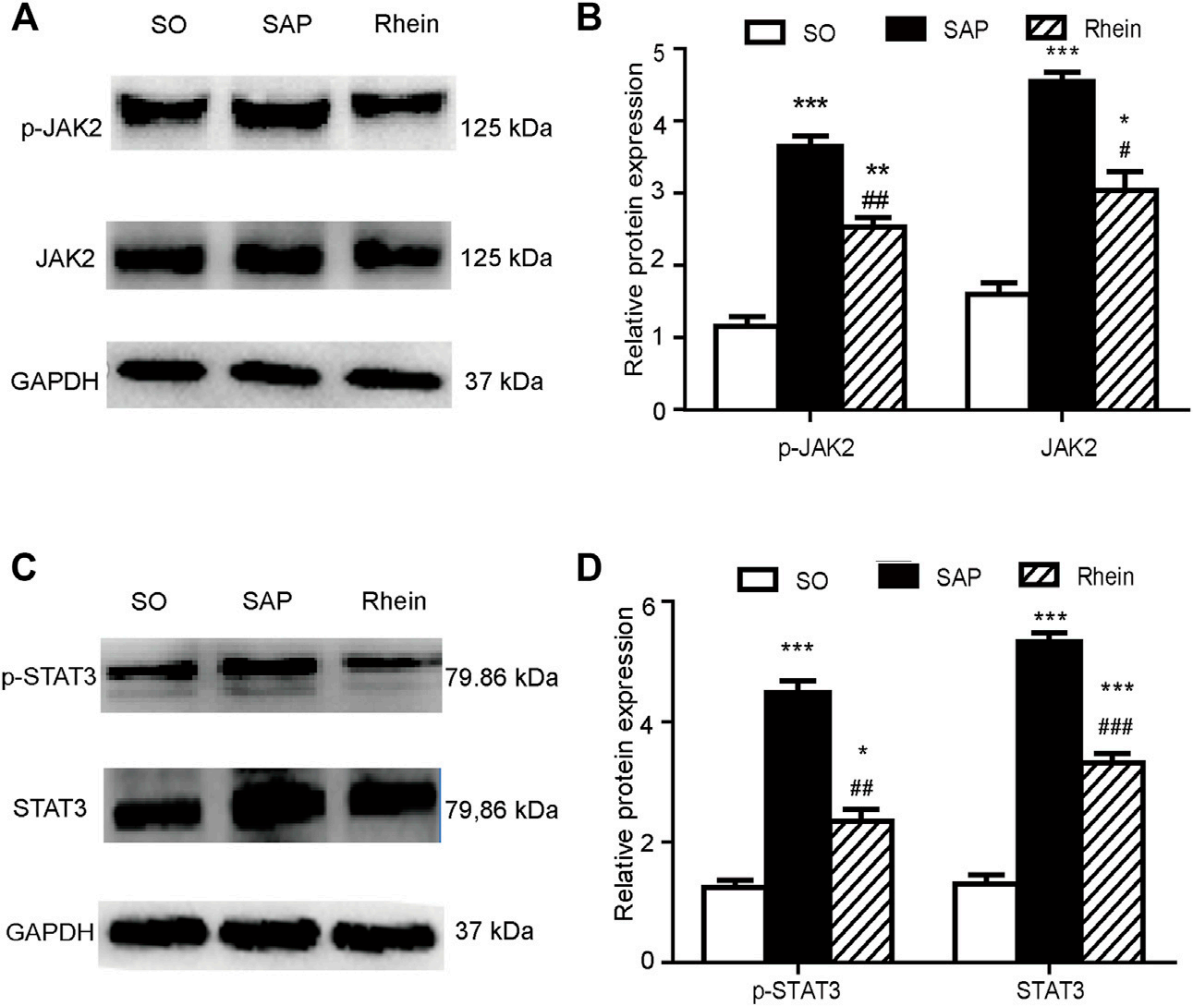
Effects of rhein on p-JAK2, p-STAT3, JAK2 and STAT3 expression in pancreatic tissue of SAP rats. **(A,B)** p-JAK2 and JAK2 protein expression in the SO, SAP and rhein groups. **(C,D)** p-STAT3 and STAT3 protein expression in the SO, SAP and rhein groups. Data are expressed as mean ± SD (*n* = 5 per group). Significance between groups was evaluated by one way analysis of variance (ANOVA) followed by a Tukey post hoc test. *p < 0.05, **p < 0.01 and ***p < 0.001 compared with the SO group; ^#^ *p* < 0.05, ^##^ *p* < 0.01 and ^###^ *p* < 0.001 compared with the SAP group. JAK2, janus kinase two; STAT3, signal transducer and activator of transcription three; SO, sham operation; SAP, severe acute pancreatitis.

Figure 5 indicates that p-JAK2 and p-STAT3 expression were significantly increased in the SAP group compared with the control group (*p* < 0.001). After rhein treatment, the activity of p-JAK2 and p-STAT3 were significantly decreased compared with the SAP group (*p* < 0.001). Figure 6 shows that p-JAK2, JAK2, p-STAT3 and STAT3 proteins expression were significantly increased in the SAP group compared with the control group (*p* < 0.001). This increasing trend was significantly reversed by treatment with rhein (*p* < 0.001). These phenomena suggest that four proteins closely related to the JAK2/STAT3 signalling pathway are activated in the pancreatic tissue of SAP rats.

### Establishment of AR42J Pancreatic Acinar Cell Injury Model Induced by Cerulein

**FIGURE 7:**
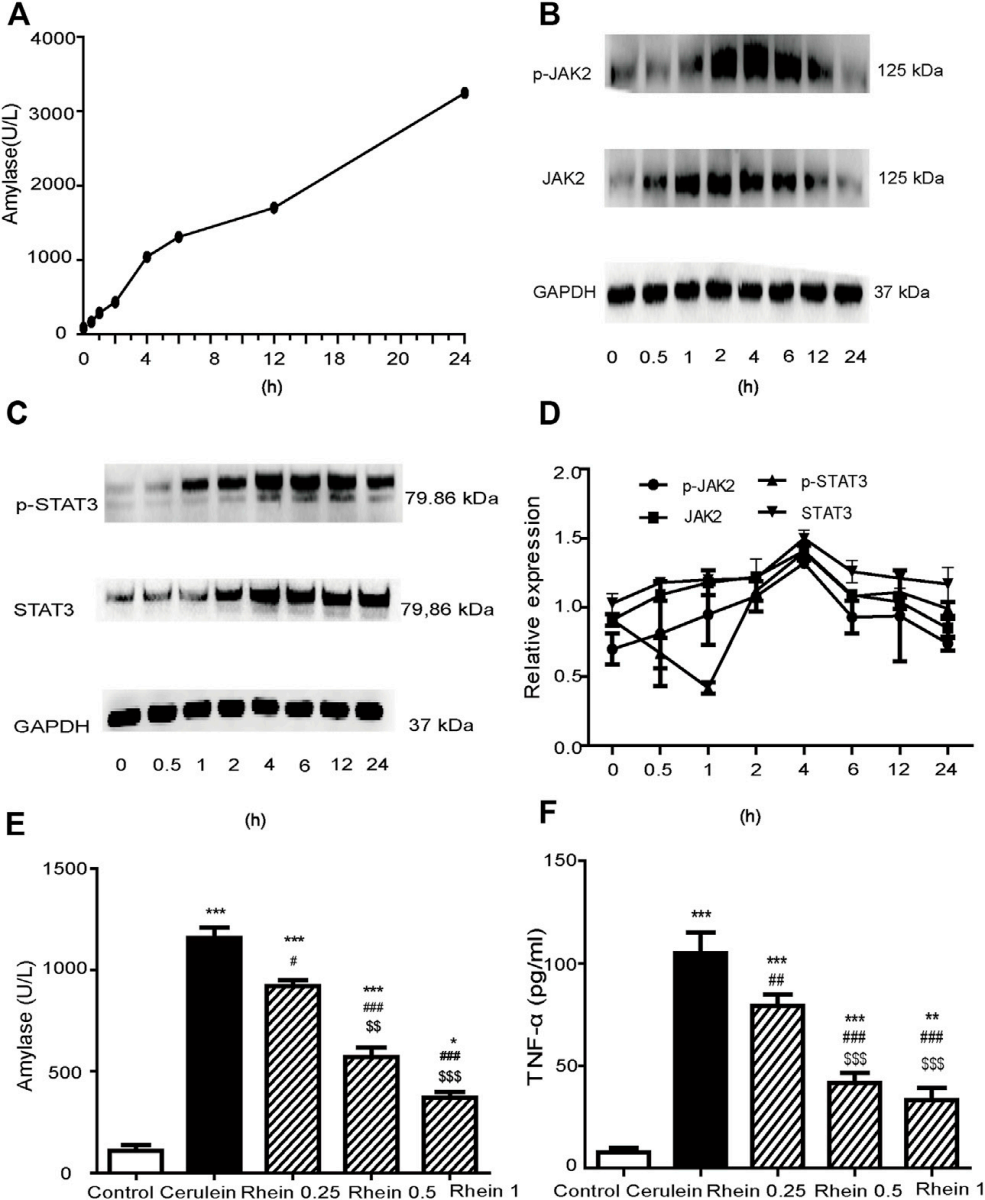
AR42J cell injury model established by cerulein treatment. **(A)** The amylase activity was measured at different time points. **(B,C)** Western blot findings of p-JAK2, JAK2, p-STAT3 and STAT3 protein expression in AR42J cells treated with cerulein. **(D)** Peak of p-JAK2, JAK2, p-STAT3 and STAT3 proteins expression at different simultaneous phase sites. **(E)** amylase activity in the control, cerulein, rhein 0.25 μg/ml, rhein 0.5 μg/ml and rhein 1 μg/ml groups **(F)** The TNF-α levels in the control, cerulein, rhein 0.25 μg/ml, rhein 0.5 μg/ml and rhein 1 μg/ml groups. Data are expressed as mean ± SD (*n* = 5 per group). Significance between groups was evaluated by one way analysis of variance (ANOVA) followed by a Tukey post hoc test. JAK2, janus kinase two; STAT3, signal transducer and activator of transcription 3.

We constructed an *in vitro* cell model mimicking SAP by treating the AR42J cells line with cerulein to stimulate pro-inflammatory cytokine expression. Figure 7A reveals that amylase activity increased significantly at 4 h, and then gradually increased at 6, 12 and 24 h. Figures 7B–D shows that JAK2, STAT3, p-JAK2 and p-STAT3 protein expression gradually increased and peaked at 4 h (*p* < 0.001), to gradually decrease at 6, 12 and 24 h. Therefore, we chose 4 h as the checkpoint for sample collection of cells in the succeeding experiments. These results suggested that the AR42J cell injury model was successfully established.

Figure 7E shows that the amylase activity in the cerulein group was significantly upregulated compared with the control group (*p* < 0.001). When rhein was added to the supernatants of cells at concentrations of 0.25, 0.5 and 1 μg/ml 20 min before adding cerulein, the amylase level downregulated in a concentration-dependent manner (*p* < 0.001). Figure 7F reveals that the TNF-α level in the cerulein group was significantly upregulated compared with the control group (*p* < 0.001). This increasing trend was significantly reversed by treatment with rhein in a concentration-dependent manner (*p* < 0.001). These results suggested that rhein protected the injured AR42J cells.

### Effect of Rhein on the AR42J Cell Injury Model

**FIGURE 8:**
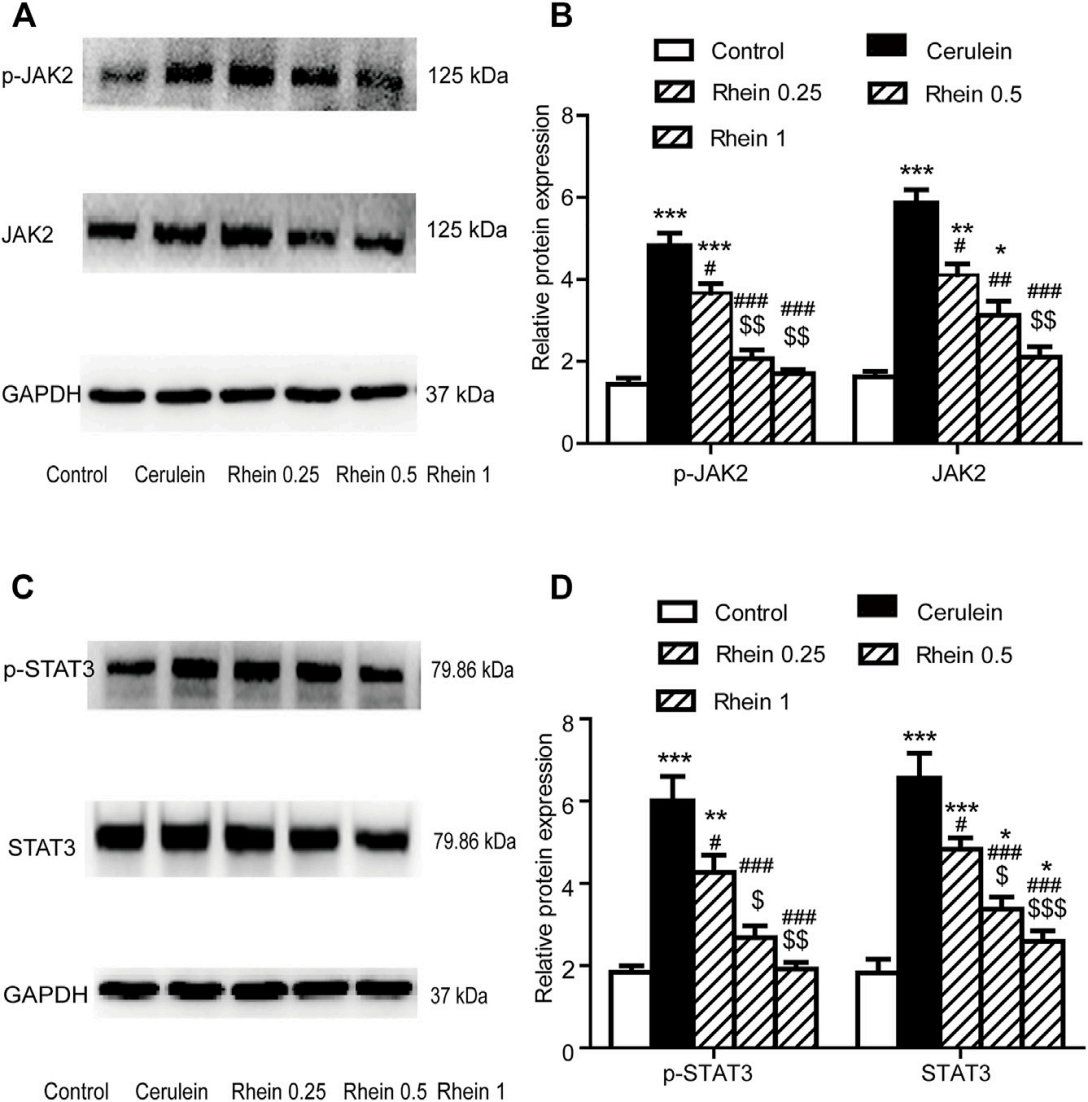
The protein expression of p-JAK2, JAK2, p-STAT3 and STAT3 in the AR42J cell model was measured by western blot. **(A,B)** p-JAK2 and JAK2 protein expression in the control, cerulein, rhein 0.25 μg/ml, rhein 0.5 μg/ml and rhein 1 μg/ml groups. **(C,D)** p-STAT3 and STAT3 protein expression in the control, cerulein, rhein 0.25 μg/ml, rhein 0.5 μg/ml and rhein 1 μg/ml groups. Data are expressed as mean ± SD (*n* = 5 per group). Significance between groups was evaluated by one way analysis of variance (ANOVA) followed by a Tukey post hoc test. *p < 0.05, **p < 0.01 and ***p < 0.001 compared with the control group; ^#^ *p* < 0.05, ^##^ *p* < 0.01 and ^###^ *p* < 0.001 compared with the cerulein group; ^$^ *p* < 0.05, ^$$^ *p* < 0.01 and ^$$$^ *p* < 0.001 compared with the rhein 0.25 μg/ml group. JAK2, janus kinase two; STAT3, signal transducer and activator of transcription 3.

Effect Of Rhein On JAK2 and STAT3 Expressions In The AR42J Cell Injury Model Figure 8 shows that p-JAK2, JAK2, p-STAT3 and STAT3 proteins expression were significantly increased in the cerulein group compared with the control group (*p* < 0.001). After rhein treatment, the activity of p-JAK2, JAK2, p-STAT3 and STAT3 were significantly decreased compared with the cerulein group (*p* < 0.001). The expression of p-JAK2, JAK2, p-STAT3and STAT3 proteins at the middle and lower doses in the rhein group differed significantly from those at the high dose. These results suggest that rhein could inhibit the expression of protein p-JAK2, JAK2, p-STAT3and STAT3, which are important components in the JAK2/STAT3 signalling transduction pathway induced by cerulein in AR42J cells.

## Discussion

In the current study, we found that rhein significantly alleviated pancreatic histopathology, attenuated proinflammation factors, and inhibited JAK2/STAT3 signalling pathway in SAP rat model. In SAP cell model, rhein significantly decreased the expression of amylase and TNF-α induced by cerulein, and inhibited JAK2/STAT3 signalling pathway.

The pathogenesis of SAP seems to be related to multifaceted pathological processes, involving inflammation, parenchymal acinar cell death by necrosis, and cellular damage in the pancreas. We established SAP rat models by retrograde pancreatic bile duct injection of sodium taurocholate (Pereda et al., 2004; Wu et al., 2013; Pérez et al., 2015). However, under the action of sodium taurocholate, pancreatic acinar cells are damaged and the inflammatory response is enhanced, which may activate various disease-related signalling pathways, such as the JAK2/STAT3 signalling pathway (Booth et al., 2011; Husain et al., 2012; Lerch and Gorelick, 2013). Conversely, crosstalk between acinar cells and the immune system perpetuates the inflammatory response. These pathways amplify the production of proinflammatory cytokines, including TNF-α, IL-1 β, IL-6 and IL-18 (Lerch and Gorelick, 2013; Abu-El-Haija and Lowe, 2018; Lee and Papachristou, 2019). The SAP cell model was induced by cerulein. Pathological changes similar to those of human SAP were found during the induction (Yu et al., 2008; Lee et al., 2010). Because cerulein is a CCK analogue, it binds to CCK receptors to activate signal transduction in pancreatic acinar cells. The CCK2 receptor is a Gq protein-coupled receptor that mediates JAK2/STAT3 activation and promotes pancreatic tumour cell proliferation (Ferrand et al., 2005; Ferrand et al., 2006; Beales and Ogunwobi, 2009). Previous studies have shown that cerulein induces IL-1β expression in pancreatic acinar cells by activating the JAK2/STAT3 signalling pathway (Yu et al., 2006; Yu et al., 2007; Zhou et al., 2021). Therefore, relieving pancreatic inflammation and inhibiting activated JAK2/STAT3 should be included in new-generation of drugs for SAP.

Traditional Chinese medicine has rich experience in the prevention and treatment of SAP. At present, it has been reported that Dachengqi Decoction plays an important role in reducing the complications of SAP in patients (Zhang et al., 2008; Wan et al., 2011). In SAP rats treated with Dachengqi Decoction and rhubarb, the distribution characteristics of five main anthraquinone compounds of rhubarb (rhein, emodin methyl ether, rhubarb phenol, aloe-emodin and emodin) in pancreas were studied, rhein is the most abundant component of all anthraquinones detected in pancreatic tissue (Zhao et al., 2004; Gong et al., 2009). However, the research on the inhibitory effect of the above traditional Chinese medicine on SAP is often limited to the determination of inflammatory factors and pharmacodynamics, and there is a lack of in-depth research on its mechanism.

At present, natural products with low side effects and efficacy have been considered for the treatment of SAP. In SAP model, huperzine II treatment was able to increase antioxidant, anti-inflammatory and anti-apoptotic activity (Piao et al., 2017), possibly by activating the farnesoid X receptor or affecting the JAK2/STAT3 signalling pathway to reduce oxidative, inflammatory and apoptotic levels (Li et al., 2020), thus providing protection for SAP. Another study also indicated that curcumin ameliorates acute renal injury in a rat model of SAP. The molecular mechanism of its effect may be associated with the suppression of the JAK2/STAT3 pathway to reduce TNF-α and IL-6 levels in SAP-induced acute renal injury. Rhein is a natural molecule and our results showed that it inhibited the JAK2/STAT3 signalling pathway, and caused a decrease in inflammatory factor levels, these results consistent with the predicted target results of network pharmacology. Besides, Rhein is widely found in medicinal plants such as rhubarb, *Sennae folium*, *Semen cassiae*, and *Polygonum multiflorum*, and it is widely used in clinical practice (Xian et al., 2020). These medical plants are broadly used all over the world. In China, approximately 10% (800) of more than 8,000 proprietary Chinese medicines contain rhubarb. So, rhein has extremely important value in the treatment of SAP.

Nowadays, rhein is widely used in inflammation treatment in China (Ji and Gu, 2021; Wang et al., 2020). In this study, in order to investigate whether rhein possessed anti-inflammatory effects in a rat model of SAP, we first established a rat model of SAP. In our current study, SAP model was established in rats by retrograde pancreatic bile duct injection of sodium taurocholate with a dose of 3.5% (Pereda et al., 2004; Wu et al., 2013; Pérez et al., 2015). Then the model was tested by pathological damage and inflammatory cytokines. For pancreatic injury, we tested it by HE staining. Edema, necrosis and inflammatory infiltration were observed in the acinar cells of pancreatic tissue. These alterations can be attributed to the action of sodium taurocholate. However, under the action of sodium taurocholate, pancreatic acinar cells are damaged and the inflammatory response is enhanced, which may activate various disease-related signalling pathways, such as the JAK2/STAT3 signalling pathway (Booth et al., 2011; Husain et al., 2012; Lerch and Gorelick, 2013). Previous studies have reported that the JAK2/STAT3 pathway plays a key role in inflammatory diseases (Zhu et al., 2017; Xia et al., 2021). Therefore, the JAK2/STAT3 signalling pathway plays a crucial role in evaluating potential mechanism. In this work, p-JAK2, JAK2, p-STAT3 and STAT3 proteins expression were significantly increased in the SAP group compared with the control group. The above-mentioned findings indicated that a SAP rat model was successfully established.

After rhein treatment, inflammatory cytokines were decreased, and JAK2/STAT3 signalling pathway related proteins were also downregulated, suggesting that rhein plays an anti-inflammatory role by inhibiting JAK2/STAT3 signaling pathway. In addition, HE staining is another method frequently used in evaluation of pancreatic injury (Wang et al., 2020). In the current study, the protective effects of rhein were further validated by the significantly improved histological results. The histopathological changes were significantly restored by rhein treatment, and less infiltration of inflammatory cells was observed in the rhein group compared with the model group. These findings also provided convincing evidence that rhein exerted protective effects against sodium taurocholate-induced pancreatic injury.

According to published many literatures about rhein anti-inflammatory mechanism, such as the interactive relationships of rhein on multiple inflammatory signaling pathways and cellular processes (Wang et al., 2020; Ding et al., 2020) The SAP cell model was induced by cerulein in AR42J cells, which pathological changes similar to those of human SAP were found during the induction (Yu et al., 2008; Lee et al., 2010). AR42J cells were treated with 10^−8^ mol/L of cerulein for 24 h. Then the model was tested by inflammatory cytokines and JAK2/STAT3 signalling pathway related proteins at 4 h. These alterations can be attributed to the action of cerulein. In this work, amylase and TNF-α activity in the SAP group were significantly upregulated compared with the control group, and p-JAK2, JAK2, p-STAT3 and STAT3 proteins expression were significantly increased in the SAP group compared with the control group. The above-mentioned findings indicated that SAP cell model was successfully established. After rhein treatment, inflammatory cytokines were downregulated, and JAK2/STAT3 signalling pathway related proteins were also decreased, suggesting that rhein plays an anti-inflammatory role by regulating JAK2/STAT3 signaling pathway.

In summary, rhein not only inhibits the release of inflammatory factors but also alters the expression of JAK2/STAT3 signal pathway-related proteins in the SAP model *in vivo* and *in vitro*. Our findings indicate the potential therapeutic effects of rhein on SAP.

## Conclusion

Taken together, our data provided evidence that rhein plays therapeutic role by inhibiting therelease of inflammatory factors in the SAP model *in vivo* and *in vitro*. This effect may be related to the regulation of JAK2/STAT3 signal pathway. This study will be helpful for the treatment of SAP.

## Data Availability

The original contributions presented in the study are included in the article/Supplementary Material, further inquiries can be directed to the corresponding authors.
